# Comparative Analysis of Mitochondrial Genomes in Diplura (Hexapoda, Arthropoda): Taxon Sampling Is Crucial for Phylogenetic Inferences

**DOI:** 10.1093/gbe/evt207

**Published:** 2014-01-02

**Authors:** Wan-Jun Chen, Markus Koch, Jon M. Mallatt, Yun-Xia Luan

**Affiliations:** ^1^Key Laboratory of Insect Developmental and Evolutionary Biology, Institute of Plant Physiology & Ecology, Shanghai Institutes for Biological Sciences, Chinese Academy of Sciences, Shanghai, China; ^2^Biocentre Grindel and Zoological Museum, University of Hamburg, Germany; ^3^School of Biological Sciences, Washington State University

**Keywords:** monophyly of diplura, mitochondrial genomes, taxon sampling, tRNA truncation

## Abstract

Two-pronged bristletails (Diplura) are traditionally classified into three major superfamilies: Campodeoidea, Projapygoidea, and Japygoidea. The interrelationships of these three superfamilies and the monophyly of Diplura have been much debated. Few previous studies included Projapygoidea in their phylogenetic considerations, and its position within Diplura still is a puzzle from both morphological and molecular points of view. Until now, no mitochondrial genome has been sequenced for any projapygoid species. To fill in this gap, we determined and annotated the complete mitochondrial genome of *Octostigma sinensis* (Octostigmatidae, Projapygoidea), and of three more dipluran species, one each from the Campodeidae, Parajapygidae, and Japygidae. All four newly sequenced dipluran mtDNAs encode the same set of genes in the same gene order as shared by most crustaceans and hexapods. Secondary structure truncations have occurred in *trnR*, *trnC*, *trnS1*, and *trnS2*, and the reduction of transfer RNA D-arms was found to be taxonomically correlated, with Campodeoidea having experienced the most reduction. Partitioned phylogenetic analyses, based on both amino acids and nucleotides of the protein-coding genes plus the ribosomal RNA genes, retrieve significant support for a monophyletic Diplura within Pancrustacea, with Projapygoidea more closely related to Campodeoidea than to Japygoidea. Another key finding is that monophyly of Diplura cannot be recovered unless Projapygoidea is included in the phylogenetic analyses; this explains the dipluran polyphyly found by past mitogenomic studies. Including Projapygoidea increased the sampling density within Diplura and probably helped by breaking up a long-branch-attraction artifact. This finding provides an example of how proper sampling is significant for phylogenetic inference.

## Introduction

Mitochondrial genomes are popular genetic markers used in population genetics studies and phylogenetic analyses of metazoan relationships. The gene components of mitochondrial (mt) genomes are relatively constant across metazoans, mostly consisting of 13 protein-coding genes (PCGs), 22 transfer RNA (tRNA) genes, and two ribosomal RNA (rRNA) genes (Boore 1999). A large noncoding region is also present and is presumed to function in controlling the replication and translation of mitochondrial genes. In insects, this is called the A+T-rich region (Zhang et al. 1995). More than 3,000 complete mitochondria sequences of metazoans have been deposited in the public databases (http://www.ncbi.nlm.nih.gov, last accessed January 7, 2014) and provide a foundation for large-scale comparative mt genome studies. This number, however, is still far from enough, compared with the extreme species richness of metazoans, especially of arthropods. In addition, relatively few mt genomes from closely related taxa are available to investigate mitochondrial genome evolution over short time scales (Cameron et al. 2007).

Despite their frequent use, the value of mt genes in deep-phylogeny studies is hotly debated (Cameron et al. 2004; Hassanin et al. 2005) because insights inferred from these genes often conflict with those from other molecular markers, especially nuclear genes (Carapelli et al. 2007; Mallatt et al. 2010; Regier et al. 2010). On the one hand, the use of mt genes in phylogenetic analysis has some obvious advantages over nuclear genes. That is, the complete genome sequence is easy to get, the ortholog assignment is accurate, and special features of mt genomes, such as gene order, contain valuable phylogenetic information (Boore 1999). Also, the secondary structure of the RNAs contains significant phylogenetic signal (Carapelli et al. 2004). On the other hand, mt genomes evolve in complex and sometimes poorly understood ways, by “rules” that may differ among animal taxa (Hassanin 2006; Rota-Stabelli et al. 2010). This makes deep-phylogeny reconstruction difficult, even prone to error. One of the confounding factors is heterogeneity of nucleotide composition across taxa, and such compositional biases can even exist between the two strands of the same mt genome due to asymmetric replication of the mt genome. The nucleotide compositions of insect mt genomes are extensively biased toward A and T (Hassanin et al. 2005).

Diplura is a group of soil-dwelling microarthropods, with a usual body length of less than 1 cm, although a few species of the Japygoidea are up to 6 cm long (Chou and Huang 1986). There are about 1,000 described dipluran species worldwide (Koch 2009). According to the shape of the cerci, Diplura are classified into three major superfamilies: Campodeoidea (with filamentous cerci), Japygoidea (with strongly sclerotized forceps), and Projapygoidea (with short, cone-shaped cerci equipped with spinnerets) (Rusek 1982). The monophyly of Diplura was questioned mainly because ovary structures vary among the superfamilies (Štys et al. 1993), but many other morphological characteristics, as well as some molecular studies, support dipluran monophyly (Koch 1997; Luan et al. 2005; Dallai et al. 2011). So far, research on Diplura has been relatively sparse, and most phylogenetic conclusions about them are based on a very limited sampling of dipluran taxa.

Mitochondrial genomes are presently available for only two species of the Campodeoidea (Podsiadlowski et al. 2006) and for one species of the Japygoidea (Carapelli et al. 2005). With these three sequences included in phylogenetic analyses of the Pancrustacea, Carapelli et al. (2007) recovered a monophyletic Diplura only from the amino acid sequences of the 13 protein-coding mitochondrial genes, whereas the nucleotide sequences of these genes suggested dipluran polyphyly instead. More recently, Simon and Hadrys (2013) failed to recover a monophyletic Diplura with the amino acid data from the hitherto densest taxon sampling of hexapods and many other animal groups: that is, *Campodea* grouped with Collembola, whereas *Japyx* clustered with some crustaceans in their 684-taxa and 300-taxa analyses. A monophyletic Diplura was only recovered in their reduced, 100-taxa and hexapod data set but with low bootstrap values (57% and 51%, respectively). We wonder whether these conflicting results, of dipluran polyphyly versus monophyly, were caused by an inadequate sampling of diplurans, especially the lack of species from the Projapygoidea. Projapygoids are assumed to represent either the most plesiomorphic subgroup of the Diplura or an evolutionary link between Campodeoidea and Japygoidea (Rusek 1982), but few comparative studies have included projapygoid species because they are very hard to collect. The mt genome information from projapygoids could help to double-check the monophyly of Diplura and to clarify the phylogenetic position of Projapygoidea within Diplura.

The phylogenetic position of Diplura within Hexapoda is also still debated. On the basis of morphology, Hennig (e.g., Hennig 1981) founded the traditional grouping of Diplura with Protura and Collembola in a clade Entognatha (for review, see Giribet and Edgecombe 2012; Trautwein et al. 2012). Other anatomical, ultrastructural, and palaeontological studies (Kukalová-Peck 1987; Koch 1997; Dallai et al. 2011), however, favored a sister group relationship between Diplura and Insecta (also see Edgecombe 2010). Molecular studies, in contrast, indicated that Diplura is sister to Protura, especially most analyses based on 18S and 28S rRNA genes (Luan et al. 2005; Gao et al. 2008; Mallatt et al. 2010). The very recent large-scale phylogenomic studies are ambiguous about the phylogenetic position of Diplura (Meusemann et al. 2010; von Reumont et al. 2012; Dell'Ampio et al. 2014). Mitogenomic analyses that included the three available dipluran mt genomes did not even recover a monophyletic Hexapoda but suggested that some crustacean lineages are more closely related to insects than are the entognathan clades (Nardi et al. 2003; Cook et al. 2005; Carapelli et al. 2007). Whether such drastically conflicting results are due to sparse taxon sampling remains to be clarified.

In this study, we sequenced and annotated the complete mitochondrial genome of *Octostigma sinensis* (Projapygoidea), representing the highest order group of Diplura not yet sampled. We also did the same for three other dipluran mitochondrial genomes, to increase the sampling of Campodeoidea and Japygoidea (Parajapygidae and Japygidae). With seven dipluran mitogenomes now available, we performed phylogenetic analyses to test for dipluran monophyly and for the relationships among the dipluran superfamilies.

## Materials and Methods

### Taxon Sampling and Specimen Collection

*Octostigma sinensis* Xie and Yang, 1991 (Projapygoidea: Octostigmatidae) was collected in South China (Zhanjiang, Guangdong Province). *Parajapyx emeryanus* Silvestri, 1928 (Japygoidea: Parajapygidae) was from Tianping mountain (Suzhou, Jiangsu Province), which is about 100 km from Shanghai. *Occasjapyx japonicus* (Enderlein, 1907) (Japygoidea: Japygidae) was from Minhang District, Shanghai, and *Lepidocampa weberi* Oudemans, 1890 (Campodeoidea: Campodeidae) was from Shanghai Botanic Garden. All specimens were morphologically identified and kept alive in a humid incubator for a short time before DNA extraction.

### Mitochondrial Genome Sequencing and Assembly

The total DNA was extracted from one specimen per species, using the commercial kit Wizard SV Genomic Purification System (Promega) following the manufacturer's instructions, and then used as the template for polymerase chain reaction (PCR) amplifications. The general strategy for amplification and sequencing was first to amplify short fragments of mitochondrial genes using universal primers (Simon et al. 2006), which were slightly modified at the degenerate sites according to the three published dipluran mt genome sequences (Carapelli et al. 2005; Podsiadlowski et al. 2006). Then, species-specific primers were designed from the sequenced fragments to amplify the long overlapped regions. The PCR conditions for short fragments using Tiangen Taq Mix are as follows: 94 °C for 4 min, 35 cycles of 94 °C for 1 min, annealing at 48–60 °C for 1 min, extension at 72 °C for 1–4 min, and a final extension at 72 °C for 10 min (annealing temperature and extension time varied with different primer pairs and targeted fragment sizes). The long fragments, using the species-specific primers, were amplified by two-step PCR using LA taq (TaKaRa, Dalian) and the conditions as described in Chen et al. (2011). The short amplified products (smaller than ∼1,500 bp) were sequenced using the amplification primers. The longer products were sequenced using primer walking. All sequencing was done by a local commercial sequencing service (Sangon Biotech, Shanghai). A small number of PCR products that could not be sequenced directly, because they had complex secondary structures or high A + T content, were cloned into the PMD-19T vector (TaKaRa, Dalian), then transformed to JM109 competent cell (TaKaRa, Dalian), and sequenced using M13 primers. All sequencing reads were assembled with the program Seqman in the DNASTAR package (Burland 2000). The accuracy of the assembly was checked manually.

### Annotation and Bioinformatics Analysis

The assembled consensus sequence of each dipluran mtDNA was further annotated and analyzed, by the following steps: 1) preliminary annotation by DOGMA (Wyman et al. 2004) provided overall information on mt genomes. 2) The tRNA genes were found by comparing the results predicted from the programs tRNAscan-se (Lowe and Eddy 1997), ARWEN (Laslett and Canback 2008), and DOGMA (Wyman et al. 2004) based on structure information. We referred to figure 4 of Podsiadlowski et al. (2006) to draw the *trnR* for *L. weberi*. 3) PCGs were identified as open reading frames, from alignments of homologous genes of the seven diplurans, which were performed with BioEdit (version 7.0.1) (Hall 1999) and DAMBE (version 5.1.1) (Xia and Xie 2001). Blast searches in National Center for Biotechnology Information (NCBI) also helped to identify and annotate the PCGs. 4) Based on known gene-order information, the boundaries of the 16S rRNA (*rrnS*) gene were assumed to be delimited by the ends of the *trnV-trnL1* pair. The 12S rRNA (*rrnL*) gene was assumed to start from the end of *trnV*, and its end was roughly identified by alignment with the three published dipluran sequences. Gene length, nucleotide composition, codon usage of the 13 PCGs, and RNA secondary structure were compared among the seven dipluran mt genomes. Nucleotide frequencies and codon usage were determined by MEGA (version 5.05) (Tamura et al. 2011). In arthropods, the two DNA strands of mitochondria are referred to as the majority strand (J-strand), on which more genes are coded, and the minority strand (N-strand). The AT and GC skews were calculated for the J-strand (all positions), the J-strand oriented and N-strand oriented PCGs, and the first, second, and third codon positions of J-strand and N-strand oriented PCGs separately. The calculating formulae are AT skew = (A−T)/(A + T) and GC skew = (G−C)/(G + C) (Perna and Kocher 1995).

### Sequence Alignment

Complete mt genome sequences of 74 relevant taxa were retrieved from the NCBI database, including 49 hexapods, 19 crustaceans, 2 myriapods, 3 chelicerates, and 1 onychophoran as the nonarthropod outgroup. These cover all four of the classical subphyla of arthropods, with a focus on the pancrustacean clade. Together with our new data on four more dipluran mt genomes, and the mt genes of the proturan *Acerentomon franzi* that were assembled from EST sequences (*nad4L* gene not found) (Meusemann et al. 2010), a total of 79 taxa was initially included in the phylogenetic analysis. Species details are listed in supplementary table S1 in supplementary file S1, Supplementary Material online.

The nucleotide sequences of each PCG were retroaligned based on the conservation of translated amino acids using DAMBE version 5.1.1 (Xia and Xie 2001). Each alignment was trimmed with the program Gblocks by Condons (version 0.91b, Talavera and Castresana 2007). All 13 trimmed alignments were concatenated as a final alignment of 9,435 nt positions. Then, the nucleotide data set was translated into the corresponding amino acid sequences, resulting in an alignment of 3,145 amino acid positions.

To add more phylogenetic signal, the nucleotide sequences of the genes for 12S rRNA and 16S rRNA were also aligned and added to the amino acid and protein-nucleotide alignments. These rRNA genes were available for 76 of the 79 taxa (unavailable for two of the collembolans, *Onychiurus orientalis* and *Podura aquatica*, and the proturan *A. franzi*)*.* Each rRNA gene was prealigned with MAFFT (version 7.027: Katoh et al. 2005) using default parameters and the strategy of “-auto” and was then realigned with RNAsalsa 0.8.1 (Stocsits et al. 2009) with the secondary structure of the insect *Apis mellifera* as the constraint file (provided with the program). Gblocks was then used to help remove unreliably aligned regions (Talavera and Castresana 2007). The concatenated alignment of the two trimmed rRNA genes yields 1,267 nt positions. The corresponding alignment positions for the three species that lack rRNA gene data were assigned with gaps.

### Data Partitioning

The best data partitioning schemes were sought using PartitionFinder (version 1.1.1, Lanfear et al. 2012; Leavitt et al. 2013). For amino acid data, the input alignment was predefined to 13 data blocks corresponding to the 13 PCGs. The “PartitionFinderProtein.py” was used to find the best-fit scheme, with parameters: branchlengths = “linked,” models = “all_protein,” model_selection = “BIC,” search = “greedy.” The best partitioning scheme was found to be (*atp6*, *cox1*, *cox2*, *cox3*, *cytb*) (*atp8*, *nad2*, *nad3*, *nad6*) (*nad1*, *nad4*, *nad4L*, *nad5*). A perl script (ProteinModelSelection.pl, written by Alexandros Stamatakis, the author of RAxML) was used to find the most appropriate model to run in RAxML for each partition.

For nucleotide data, the input alignment was predefined to 28 data blocks, corresponding to first and second codon position of each of the 13 PCGs, plus the two rRNA genes. The “PartitionFinder.py” was used to find the best-fit scheme for these nucleotide data. The best scheme had these eight partitions: (*atp6*_pos1, *atp8*_*pos*1, *nad2*_pos1, *nad3*_pos1, *nad6*_pos1) (*atp6*_pos2, *cox2*_pos2, *cox3*_pos2, *cytb*_pos2) (*atp8*_pos2, *nad2*_pos2, *nad3*_pos2, *nad6*_pos2) (*cox1*_pos1, *cox2*_pos1, *cox3*_pos1, *cytb*_pos1) (*cox1*_pos2) (*nad1*_pos1, *nad4*_pos1, *nad4L*_pos1, *nad5*_pos1) (*nad1*_pos2, *nad4*_pos2, *nad4L*_pos2, *nad5*_pos2) (*rrnS*, *rrnL*).

Finally, the nucleotide data of the two rRNA genes in one partition were joined with the partitioned amino acid data of the 13 PCGs as well.

### Phylogenetic Analyses

Maximum-likelihood (ML) tree searches based on amino acid sequences plus rRNA gene nucleotides (rDNAs) were carried out via the online CIPRES web portal using RAxML 7.6.3 (Stamatakis et al. 2008; Miller et al. 2010). We used RAxML rapid bootstrapping (100 replicates) and subsequent ML search, under the PROTGAMMA + MTART model for the “*atp6*, *cox1*, *cox2*, *cox3*, *cytb*” partition, the PROTGAMMA + MTZOAF model for the “*atp8*, *nad2*, *nad3*, *nad6*” and “*nad1*, *nad4*, *nad4L*, *nad5*” partitions, and the GTR + GAMMA model for the two rDNAs. The models were defined in the partition file. Bootstrap values above 60% are considered significant support.

**Fig. 1.—:**
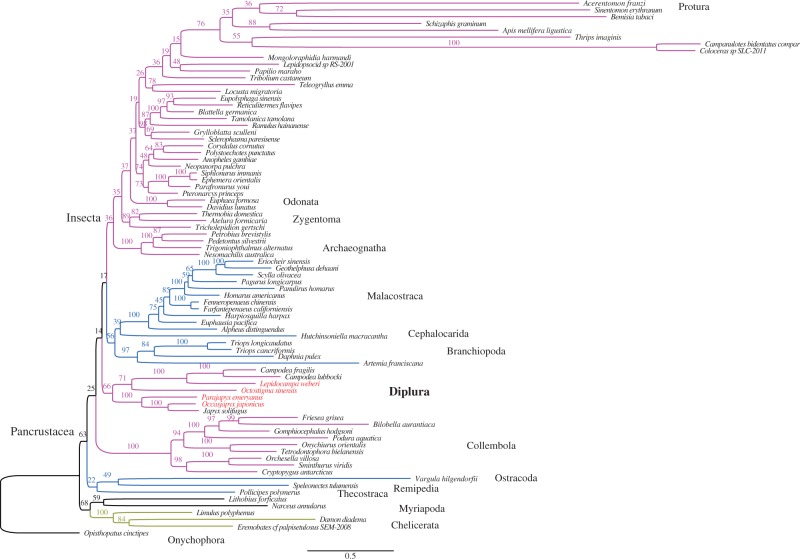
Phylogenetic tree of the complete taxa set (79 taxa) obtained from maximum likelihood estimation with amino acid data from the 13 PCGs plus rDNA sequence alignments. Species names that are printed in dark red are newly sequenced in this study.

After the first analysis, we found that eight unrelated taxa with astonishingly long branches ( = highly divergent sequences) were joined together by possible long-branch attraction (LBA) artifacts (see Discussion). However, these sequences (those on the top of fig. 1) did not influence the positions of all diplurans, so we deleted them in the subsequent analysis. With this, 71 taxa was our usual starting point.

The RAxML analyses were also carried out for the nucleotide-only data set, with the first and second codon of PCGs plus rDNA, in separate unpartitioned and partitioned trials, under the GTR + GAMMA model. RY-coding analyses, which recode the purines as R and the pyrimidines as Y for dealing with base-compositional heterogeneity, were also carried out. First, the third-codon positions of PCGs were RY coded, whereas the first and second codon positions were kept as nucleotides. We call this nt3 RY coding. Then, we RY coded both the first and third codon positions and kept the second codon positions as nucleotides (nt13 RY coding: after Delsuc et al. 2003). The RY-coded data were analyzed under the BIN + GAMMA model in RAxML.

With the unpartitioned and partitioned, nt3 RY-coded, and nt13 RY-coded, nucleotide data, we explored the effects of taxon sampling on the phylogeny of Diplura. We did so by performing phylogenetic analyses based on six different data sets: 1) 71 taxa, including all seven diplurans, 2) 70 taxa, including six diplurans but excluding the projapygoid *O. sinensis*, 3) 67 taxa, with only the three previously known diplurans that were used by Carapelli et al. (2007), 4) 68 taxa, including the three previous diplurans, and our newly sequenced *O. sinensis*, 5) 68 taxa, excluding all three taxa from Campodeoidea but including the other four diplurans, and 6) 68 taxa, excluding all three taxa from Japygoidea but including the other four diplurans. The partitioned data set was further tested by removing three other long-branched and potentially disruptive sequences that had been near the Diplura in the trees: of *Speleonectes tulumensis*, *Vargula hilgendorfii*, and *Pollicipes polymerus*. All trees were visualized and edited by Figtree v1.4 (http://tree.bio.ed.ac.uk/software/figtree/, last accessed January 7, 2014).

## Results

### Characteristics of Dipluran Mitochondrial Genomes

Table 1 summarizes aspects of the four new and three previously published dipluran mt genomes, including their GenBank accession numbers. Complete sequences were obtained for *O. sinensis* (15,122 bp), *P. emeryanus* (15,268 bp), and *Occ. japonicus* (15,746 bp). For *L. weberi*, on the other hand, although we have assembled all the sequencing reads into a circular consensus contig of 14,360 bp, the *trnI* was missed, and we obtained only 212 bp of the region between *rrnS* and *trnQ* (assumed to be the A + T-rich region). Judging from the high AT content, the secondary structure, and the stretches of polyT in hexapods’ A + T-rich region, we suspect that a fragment of about 500 bp was skipped in our PCR amplification of the *L. weberi* genome despite repeated attempts to amplify and clone this region.

**Table 1:** Characteristics of Seven Dipluran Mitochondrial Genomes

Species	Family	GenBank Accession	Genome Length (nt)	AT%	AT Skew	GC Skew	Reference
*Campodea fragilis* (cf)	Campodeidae	NC_008233	14,965	72.56	0.06	−0.29	Podsiadlowski et al. (2006)
*Campodea lubbocki* (cl)	Campodeidae	NC_008234	14,974	74.81	0.01	−0.3	Podsiadlowski et al. (2006)
*Lepidocampa weberi* (lw)	Campodeidae	JN990601	>14,360^a^	>66.73	0.06	−0.38	This study
*Octostigma sinensis* (os)	Octostigmatidae	JN990598	15,122	68.32	0.04	−0.39	This study
*Parajapyx emeryanus* (pe)	Parajapygidae	JN990599	15,268	64.92	0.18	−0.28	This study
*Occasjapyx japonicus* (oj)	Japygidae	JN990600	15,746	59.42	0.2	−0.28	This study
*Japyx solifugus* (js)	Japygidae	NC_007214	15,785	64.82	0.19	−0.29	Carapelli et al. (2005)

^a^A fragment of about 500 bp is assumed to have been skipped in our PCR amplification and sequencing process.

The genome lengths of the three campodeid species are less than 15,000 bp, whereas those of *O. sinensis* and three japygoid species are greater than 15,000 bp. That of *O. sinensis* is slightly smaller than those of the three japygoid species.

The AT contents of the campodeid species are greater than those of *O. sinensis* and the three japygoids. The actual AT content of *L. weberi* should be greater than the recorded 66.73% due to the missing part of the A + T-rich region, which usually has a very high AT content in campodeids (e.g., the AT contents of the A + T-rich region of *Campodea fragilis* and *Campodea lubbocki* are 84.23% and 89.37%, respectively). The AT-skew values of the J-strand for the campodeid species and *O. sinensis* are very low (0.01–0.06), whereas those for the three japygoid species are relatively greater (0.18–0.20). All seven dipluran mt genomes have similar GC-skews for the whole J-strand (−0.39 to −0.28) (table 1). Close examination of the skew values for genes oriented on J-strand and N-strand shows that the nucleotide compositions of the N-coded PCGs are more biased than those of the J-coded PCGs (supplementary table S2 in supplementary file S1, Supplementary Material online). Such skew-asymmetry might be caused by differential mutational bias between two strands, due to asymmetry replication of these strands (Hassanin et al. 2005).

All four of our newly sequenced mt genomes were found to comprise the same gene set as in the three previously reported diplurans, and the genes are arranged in the same order as in typical pancrustacean mt genomes. This order is listed from top to bottom in table 2, left column. Twenty-four genes are encoded by the J-strand, and 13 genes are encoded by the N-strand. The start and stop codons of each PCG, the size of each gene, and of the intergenic gaps are also given in table 2. All the PCGs start with the typical ATN codon, except that the start codon for the *cox1* of *P. emeryanus* and *Occ. japonicus* is TTA, for the *nad5* gene of *O. sinensis* and *P. emeryanus* is TTG, and of *Occ. japonicus* is GTG. These exceptions are indicated in boldface in table 2. The PCGs are terminated by either the complete (TAA or TAG) or incomplete stop codons (TA-, T-), which are presumably polyadenylated after transcription to form the complete stop codon TAA (Ojala et al. 1980). As indicated in the “Size” column of the table, homologous genes are of similar sizes among the seven diplurans. *Nad5* is the largest at over 1.7 kb, and the tRNA genes are the smallest, ranging from 52 to 71 bp. In at least one dipluran, *trnC*, *trnR*, *trnS-gcu* (*trnS1*), or *trnS-uga* (*trnS2*) is notably smaller than its counterpart in other metazoan mt genomes and was found to have a truncated secondary structure (marked in boldface in table 2 and discussed more later). The sizes of the intergenic regions are more variable, although usually small, and are only conserved across all diplurans at the junction of *nad4/nad4L* (7 bp) and *nad6/cob* (1 bp). In *C. fragilis*, there is a uniquely large noncoding region of 111 bp between *nad2* and *trnW*, a location that is relatively near the A + T-rich region (Podsiadlowski et al. 2006). Turning to the A + T-rich region itself, those of the japygoids *Occ. japonicus* and *Japyx solifugus* are 1,178 and 1,052 bp, respectively, which is larger than those of the other diplurans, and the entire mitochondrial genomes of the two japygoid species are indeed the largest among the seven diplurans (tables 1 and 2).

**Table 2:** Gene Comparison of Codons, Sizes, and Intergenic Spacers/Overlaps among Seven Dipluran mt Genomes

Gene	Strand	Start Codon							Stop Codon							Size (bp)							Intergenic (bp)						
		cf	cl	lw	os	pe	oj	js	cf	cl	lw	os	pe	oj	js	cf	cl	lw	os	pe	oj	js	cf	cl	lw	os	pe	oj	js
*trnI-gau*	+															62	61	**?**	66	64	63	63	−3	7	**?**	−3	−3	1	6
*trnQ-uug*	−															65	64	66	67	69	69	60	2	20	3	−1	4	2	−1
*trnM-cau*	+															64	64	63	65	65	64	64	0	0	0	0	0	0	0
*nad2*	+	ATA	ATT	ATA	ATG	ATG	ATG	ATG	TAA	TAA	TAA	TAA	TAA	TAA	TAA	1,005	1,005	996	1,011	1,014	1,008	1,008	**111**	3	−2	−2	6	−2	−2
*trnW-uca*	+															64	66	63	68	68	67	67	3	0	−1	−8	0	−1	−1
*trnC-gca*	−															62	**53**	62	61	61	61	62	−1	0	4	0	1	−1	−2
*trnY-gua*	−															63	62	63	64	66	62	65	−8	−5	−5	−3	−5	−5	−9
*cox1*	+	ATT	ATA	ATA	ATT	**TTA**	**TTA**	ATC	T–	TAA	TAA	T–	TAA	TAA	TAA	1,540	1,542	1,542	1,534	1,539	1,539	1,545	0	−4	−5	0	3	0	0
*trnL-uaa*	+															64	63	61	62	63	65	62	0	1	0	0	0	−1	0
*cox2*	+	ATA	ATA	ATT	ATG	ATG	ATG	ATA	T–	TAA	T–	T–	T–	T–	T–	679	684	682	682	679	679	679	0	−6	0	0	0	0	0
*trnK-cuu*	+															57	68	64	68	68	69	70	8	−3	−2	−2	0	−1	−1
*trnD-uau*	+															63	61	64	65	65	63	63	0	0	0	0	0	1	0
*atp8*	+	ATT	ATC	ATC	ATC	ATT	ATT	ATA	TAA	TAA	TAA	TAA	TAA	TAA	TAA	156	156	156	159	159	159	156	−7	−7	−7	−7	−4	−7	−4
*atp6*	+	ATG	ATG	ATG	ATG	ATA	ATG	ATA	TAA	TAA	TAA	TAA	TAA	TAA	TAA	675	675	675	672	675	678	672	4	3	3	−1	−1	3	4
*cox3*	+	ATG	ATG	ATG	ATG	ATG	ATG	ATG	TAA	T–	T–	TAA	TAA	T–	T–	792	787	787	789	789	787	787	−5	0	0	−1	−1	0	0
*trnG-ucc*	+															59	60	60	65	63	61	62	−3	0	1	0	0	0	−3
*nad3*	+	ATA	ATC	ATG	ATC	ATC	ATT	ATA	TAA	TA-	TAG	TAG	TAG	T–	T–	357	347	354	354	354	352	355	−2	2	−2	−2	−2	0	0
*trnA-ugc*	+															62	60	60	64	61	62	62	0	0	−1	5	0	2	1
*trnR-ucg*	+															**52**	**53**	**53**	62	69	60	62	−3	−3	−1	6	4	−1	−3
*trnN-guu*	+															62	61	64	64	66	62	64	−2	−1	−3	0	0	0	−5
*trnS-gcu*	+															**54**	**55**	**54**	**68**	**67**	**66**	**62**	−1	−1	−1	0	4	−1	3
*trnE-uuc*	+															64	61	62	65	63	64	65	−2	0	0	−1	15	0	−1
*trnF-gaa*	−															61	60	61	65	66	62	63	0	0	0	−8	−1	0	0
*nad5*	−	ATA	ATC	ATT	**TTG**	**TTG**	**GTG**	ATA	TAA	TAG	T–	TAA	TAA	T–	T–	1,707	1,710	1,708	1,713	1,734	1,732	1,726	−1	0	0	0	1	0	7
*trnH-gug*	−															64	60	62	64	64	62	60	−15	−1	0	0	1	1	0
*nad4*	−	ATG	ATG	ATG	ATG	ATG	ATG	ATG	TAA	TA–	T–	T–	TAA	TAA	T–	1,338	1,328	1,333	1,330	1,344	1,344	1,345	−**7**	−**7**	−**7**	−**7**	−**7**	−**7**	−**7**
*nad4L*	−	ATG	ATG	ATG	ATT	ATG	ATG	ATG	TAA	TAG	TAA	TAA	TAA	TAA	TAA	285	285	288	288	288	294	294	8	5	5	2	5	2	2
*trnT-ugu*	+															60	61	60	66	61	63	63	0	0	−1	0	0	0	−1
*trnP-ugg*	−															67	63	61	65	63	63	62	2	2	1	2	2	2	2
*nad6*	+	ATT	ATT	ATT	ATA	ATA	ATC	ATC	TAA	TAA	TAA	TAA	TAA	TAA	TAA	510	525	507	504	510	510	510	−**1**	−**1**	−**1**	−**1**	−**1**	−**1**	−**1**
*cob*	+	ATG	ATG	ATG	ATG	ATG	ATG	ATG	TAA	TAA	TAA	T–	T–	TAA	T–	1,143	1,140	1,137	1,132	1,132	1,134	1,132	−2	−2	−2	0	0	−2	0
*trnS-uga*	+															**56**	**55**	**54**	63	65	66	66	56	79	29	4	21	2	3
*nad1*	−	ATT	ATA	ATT	ATT	ATT	ATG	ATG	TAA	TAA	TAA	T–	TAG	TAG	TAG	924	921	921	952	933	936	936	0	0	12	3	15	21	39
*trnL-uag*	−															63	59	62	65	62	66	64	0	0	0	0	0	0	0
*16S rRNA*	−															1,092	1,096	1,066	1,169	1,289	1,249	1,417	1	0	0	0	0	0	0
*trnV-uac*	−															62	61	60	65	71	65	69	0	0	0	0	0	0	0
*12S rRNA*	−															722	740	700	773	764	755	742	**558**	**621**	**>212**	**668**	**578**	**1,178**	**1,052**

Note.—Species names are abbreviated as cf, *Campodea fragilis*; cl, *C. lubbocki*; lw, *Lepidocampa weberi*; os, *Octostigma sinensis*; pe, *Parajapyx emeryanus*; oj, *Occasjapyx japonicus*; js, *Japyx solifugus*. Genes are listed in the order in which they occur in the genomes. In the “Strand” column, “+” means the majority (J) strand and “−” means the minority (N) strand. Bold type marks the exceptions to typical ATN start codons in the “Start codon” column and also marks the size of notably reduced tRNAs in the “Size (bp)” column. In the “Intergenic (bp)” column, the negative numbers mean overlap between adjacent genes. Bold type marks several other features in this column: a uniquely large noncoding region of 111 bp at the junction of *nad2*/*trnW-uca* in *C. fragilis*; the 7 bp between *nad4* and *nad4L* and 1 bp between *nad6* and *cob*, which are conserved in size across the seven diplurans; and the large intergenic spacer between *12S rRNA* and *trnI-gau* (the last line) is the A + T-rich region.

### Phylogenetic Analysis of the Amino Acids Plus rDNAs

**Fig. 2.—:**
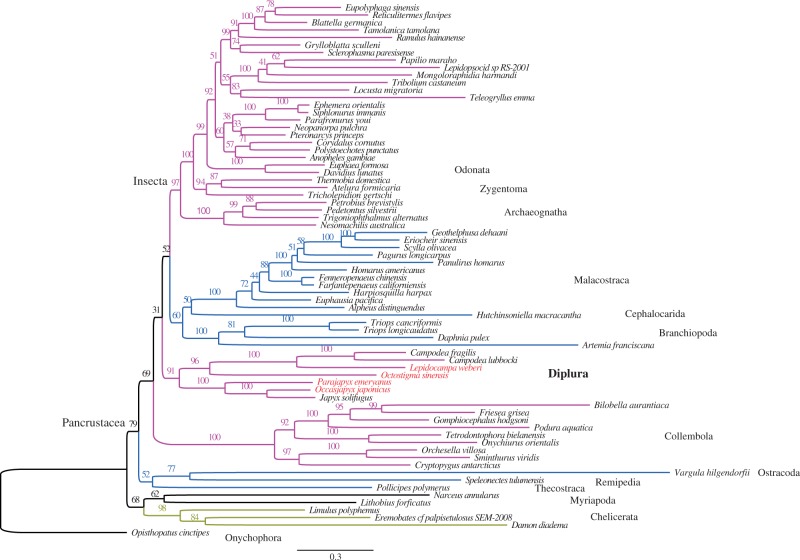
Phylogenetic tree of the reduced taxa set (71 taxa) obtained from maximum likelihood estimation with amino acid data from the 13 PCGs plus rDNA sequence alignments. The eight species with long branches at the top of the tree in figure 1 were excluded from the analysis.

The 79-taxa phylogenetic tree calculated from the complete data set, of protein amino acids plus rDNA nucleotides, supports the monophyly of Diplura, shows monophyly of Pancrustacea but does not recover a monophyletic Hexapoda (fig. 1). In this tree, Diplura and Collembola appear less closely related to insects than do the crustacean clades Malacostraca, Cephalocarida, and Branchiopoda. The two proturan species *A. franzi* and *Sinentomon erythranum* cluster among insects with the similarly long-branched sequences of hemipterans, thysanopterans, phthirapterans, and hymenopterans. This is likely an LBA artifact, and proturan mtDNAs do show very biased nucleotide compositions (Chen et al. 2011). After excluding the eight longest-branched taxa from the analysis, we obtained the tree of figure 2, which still splits the hexapods. Reducing the taxa number from 79 (fig. 1) to 71 (fig. 2) had little effect on the arrangement of the pancrustacean clades, but the bootstrap values of some nodes increased greatly (for Diplura from 66% to 91%, for Insecta from 36% to 97%). In addition, we got similar tree topologies for Pancrustacea when removing the onychophoran and myriapods, and using chelicerates as the outgroup (data not shown).

The clades recovered as monophyletic from the amino acid data plus rDNAs (figs. 1 and 2) include Diplura, Chelicerata, Collembola, Malacostraca, Branchiopoda, Archaeognatha (=Microcoryphia), and Zygentoma. Because we focus on the question of dipluran monophyly, we did not further examine the relationships within other taxa such as higher insects or crustacean subgroups.

The key result of this exercise in pan-arthropod tree reconstruction is that a monophyletic Diplura is always retrieved in our analyses based on amino acid data plus rDNAs (figs. 1 and 2).

### Phylogenetic Analysis of the Nucleotide Data Set of PCGs Plus rDNAs

**Fig. 3.—:**
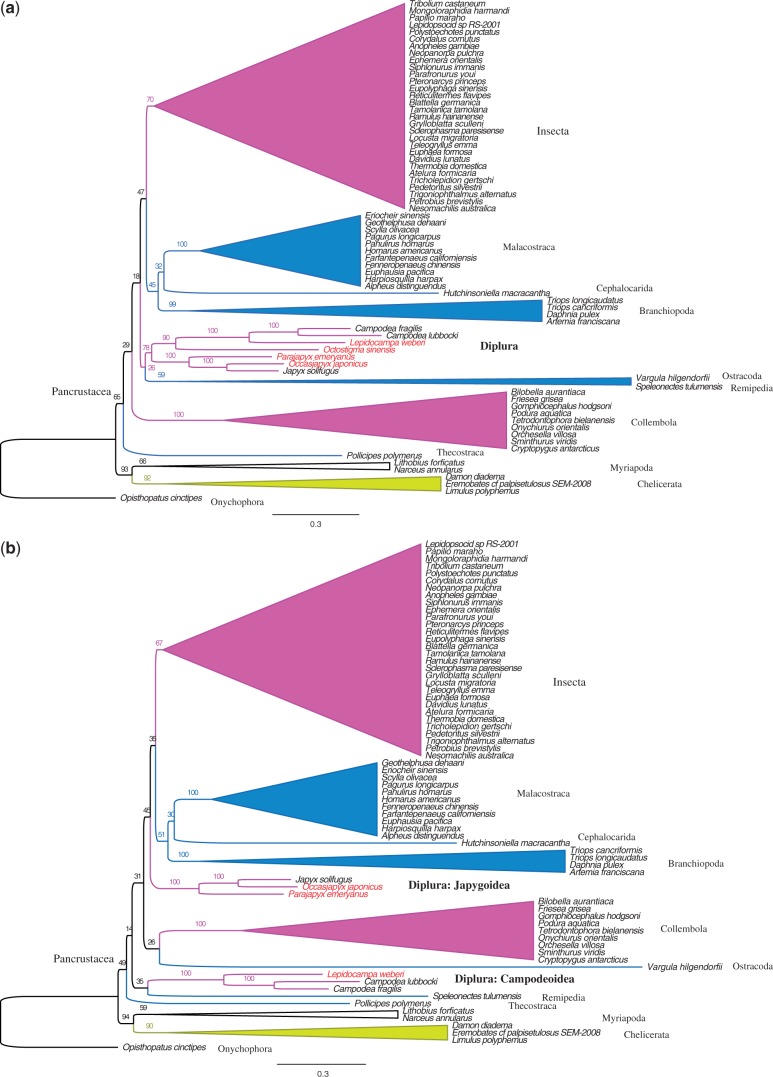

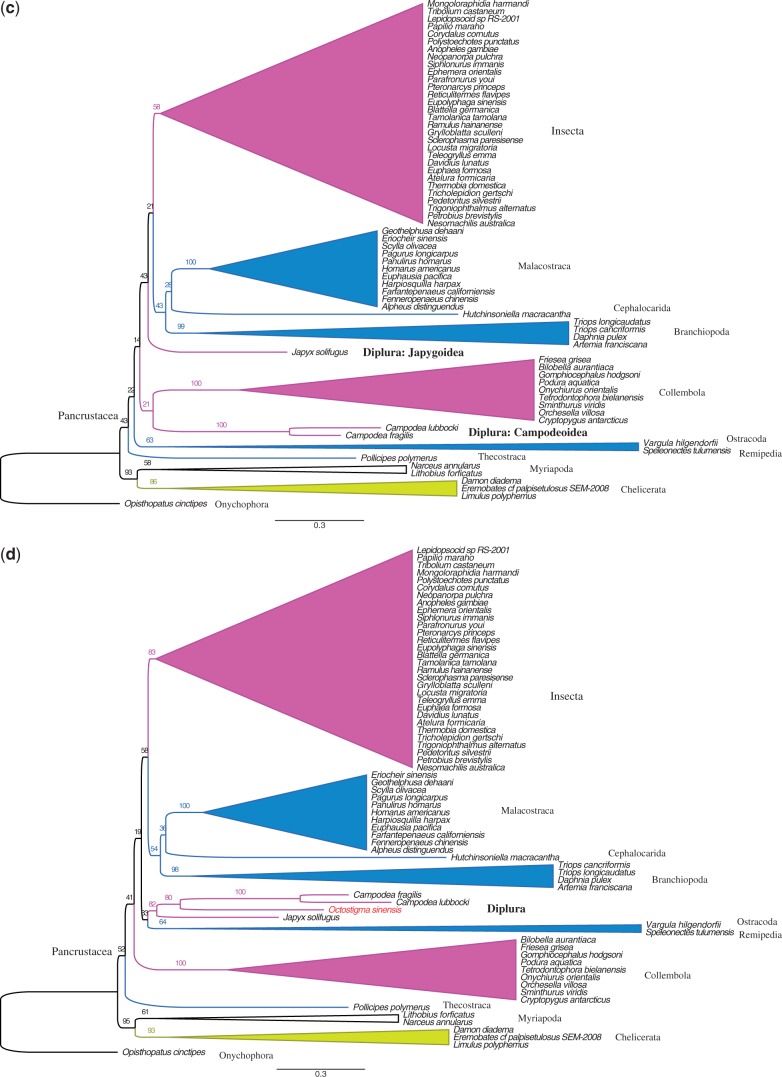

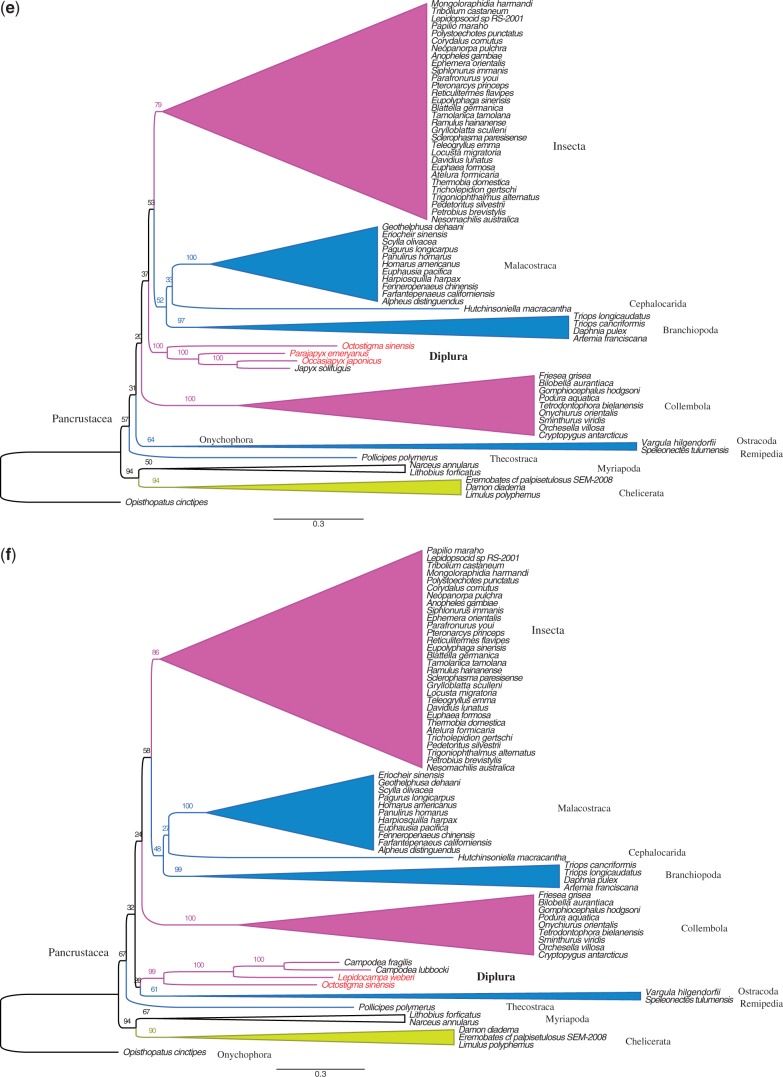
Maximum likelihood trees of nucleotide data set of PCGs plus rDNA sequence alignment under different dipluran taxon sampling. Third-codon positions were RY coded. (*a*) Tree from the data set with 71 taxa. (*b*) Data set with 70 taxa: exclusion of the projapygoid *Octostigma sinensis*. (*c*) Data set with 67 taxa: exclusion of all four new dipluran mt genomes obtained in our study. (*d*) Data set with 68 taxa: inclusion of only *O. sinensis* with the dipluran sample of Carapelli et al. (2007) (i.e., two campodeid species and one japygid species). (*e*) Data set with 68 species: all three species of Campodeoidea were excluded. (*f*) Data set with 68 species: all 3 species of Japygoidea were excluded. Complete tree topologies are provided in supplementary figure S3*A–F* in supplementary file S2, Supplementary Material online.

Next, while continuing to leave out the eight taxa with long branches, we partitioned the first and second codon positions of 13 PCGs plus rDNA into eight partitions (see Data partitioning in Materials and Methods section). The third codons were RY coded. The resulting 71-taxa tree shows a monophyletic Diplura, with 78% support (see fig. 3*a* and a more detailed version of the tree in supplementary fig. S3*A* in supplementary file S2, Supplementary Material online). Interestingly, monophyly of Diplura was lost after exclusion of the projapygoid *O. sinensis* from the analysis (70 taxa; fig. 3*b* and supplementary fig. S3*B*, Supplementary Material online). Next, exclusion of all four newly sequenced dipluran mt genomes (67 taxa) provided the result found by Carapelli et al. (2007): Campodeoidea went with Collembola, whereas Japygoidea was sister to a cluster composed of Branchiopoda, Malacostraca, Cephalocarida, and Insecta (fig. 3*c* and supplementary fig. S3*C*, Supplementary Material online). The monophyly of Diplura was recovered again just by adding the projapygoid *O. sinensis* (82% support: 68 taxa; fig. 3*d* and supplementary fig. S3*D*, Supplementary Material online). Additionally, when keeping the projapygoid species but excluding either all three campodeid species (fig. 3*e* and supplementary fig. S3*E*, Supplementary Material online) or all three japygoid species (fig. 3*f* and supplementary fig. S3*F*, Supplementary Material online), a monophyletic Diplura was always supported (100–99%). These results are summarized in table 3, in its third data column.

**Table 3:** Bootstrap Values for Diplura/Rabdura^a^ with Different Analysis Methods and Dipluran Sampling

Test Case (Dipluran Taxa Included)	71 Taxa				68 Taxa^b^	
	nt12 of 13 PCGs Plus 2 rDNAs, no RY Coding		Partitioned nt12 of 13 PCGs Plus 2 rDNAs, and nt3 RY Coded	Partitioned nt2 of 13 PCGs Plus 2 rDNAs and nt13 RY Coded	Partitioned nt12 of 13 PCGs Plus 2 rDNAs and nt3 RY Coded	Partitioned nt2 of 13 PCGs Plus 2 rDNAs and nt13 RY Coded
	Unpartitioned^c^	Partitioned				
A (all seven dipluran species)	66/77	79/86	78/90 (fig. 3*a*)	61/96 (supplementary fig. S4*A*, Supplementary Material online)	88/89	89/96
B (excluding *Octostigma sinensis* in A)	−/−	−/−	−/− (fig. 3*b*)	−/− (supplementary fig. S4*B*, Supplementary Material online)	−/−	−/−
C (only the three dipluran species studied by Carapelli et al. 2007)	−/−	−/−	−/− (fig. 3*c*)	18/− (supplementary fig. S4*C*, Supplementary Material online)	−/−	36/−
D (add *O. sinensis* in C)	78/82	88/92	82/80 (fig. 3*d*)	80/90 (supplementary fig. S4*D*, Supplementary Material online)	82/94	93/93
E (excluding all three campodeid species in A)	100/−	100/−	100/− (fig. 3*e*)	100/− (supplementary fig. S4*E*, Supplementary Material online)	100/−	100/−
F (excluding all three japygoid species in A)	100/−	100/−	99/− (fig. 3*f*)	99/− (supplementary fig. S4*F*, Supplementary Material online)	100/−	100/−

^a^Rabdura = Campodeoidea + Projapygoidea.

^b^To make the 68-taxa set, *Vargula hilgendorfii*, *Speleonectes tulumensis,* and *Pollicipes polymerus* were removed from the 71-taxa set.

^c^The “nt12 of 13 PCGs” is unpartitioned as indicated; however, the PCGs and rDNAs are defined in two different partitions.

We retested these six cases by excluding three long-branched taxa near Diplura, namely *S. tulumensis*, *V. hilgendorfii*, and *Po. polymerus*, from the 71-taxa set. This 68-taxa set was used to see whether these divergent taxa had biased the results. No such bias was indicated because bootstrap support for a monophyletic Diplura remained high, whenever the *O. sinensis* sequence was present. See the fifth data column in table 3.

In addition, we tested these six cases with both the first and third codon positions RY coded, while keeping the second codon positions as nucleotides for the 71-taxa and 68-taxa set, respectively. This demanded that we recalculate the best partition scheme, for the second codon positions of the 13 PCGs, with PartitionFinder, which gave these four partitions: (*atp6*_pos2, *atp8*_pos2, *cox2*_pos2, *cox3*_pos2, *cytb*_pos2) (*cox1*_pos2) (*nad1*_pos2, *nad4*_pos2, *nad4L*_pos2, *nad5*_pos2) (*nad2*_pos2, *nad3*_pos2, *nad6*_pos2). The results are presented in the fourth and sixth data columns of table 3 and in figures S4*A–F* in supplementary file S3, Supplementary Material online. Monophyly of Diplura was always highly supported whenever the *O. sinensis* sequence was included but was never supported (by bootstrap values over 60%) when *O. sinensis* was excluded. This further shows that the *O. sinensis* is the key to getting dipluran monophyly.

Therefore, in our phylogenetic analyses, the monophyly of Diplura was significantly supported only when the projapygoid species was included, no matter which data set was used. Table 3 also shows that our partitioned analyses, which are designed to give better results by using more realistic models of nucleotide or amino acid substitution (Simon et al. 2006; Leavitt et al. 2013), gave higher bootstrap support for dipluran monophyly than did the simpler, traditional, unpartitioned analysis. To see this, compare the first and second data columns of the table.

### The Internal Relationships of Diplura

All our analyses yielded the same relations within the Diplura (figs. 1–3*a* and table 3). Monophyly of Campodeoidea and of Japygoidea each have 100% bootstrap support, and the projapygoid *O. sinensis* consistently clusters with the Campodeoidea in the clade Rhabdura (with 77–96% support).

### Reduction of tRNA Arms

**Fig. 4.—:**
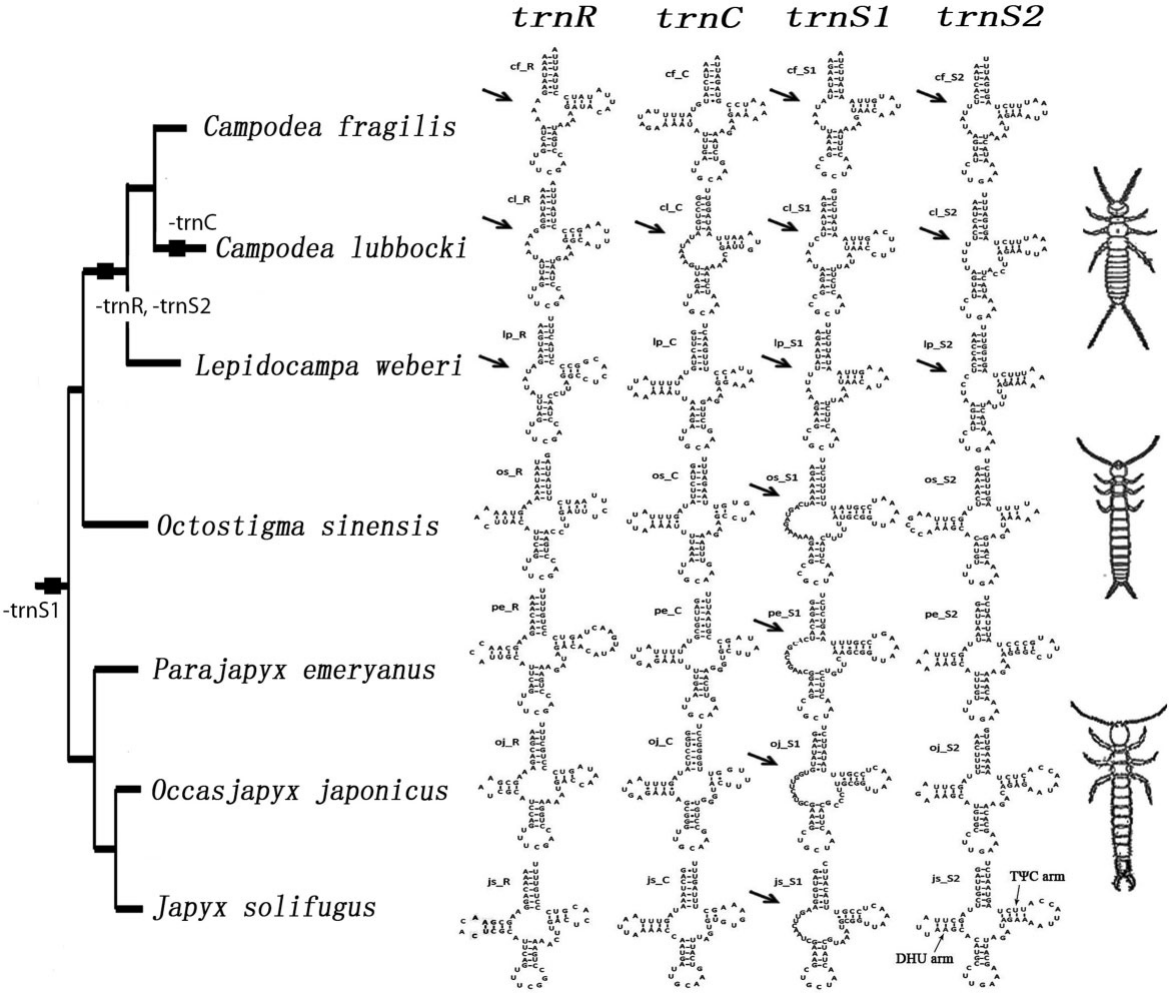
Constructed secondary structures of the mitochondrial *trnR*, *trnC*, *trnS1*, and *trnS2* mapped on the subclade of all diplurans from the tree in figure 2. Arrows indicate absence of the D-arm in tRNA molecules. The events of tRNA truncation are depicted by black squares on the nodes.

All seven dipluran mitochondrial genomes harbor the full set of 22 tRNAs, with the possible exception of *L. weberi*, where we were unable to identify *trnI* (table 2). Starting with the dipluran topology from figure 2, we marked the truncations in tRNA stems at the nodes where they occurred and thereby obtained figure 4. Loss of a tRNA arm was found for *trnR*, *trnC*, *trnS1*, and *trnS2* (fig. 4, indicated by arrows). According to our analysis based on the ARWEN program, *trnS1* lacks the dihydrouridine (DHU) arm (D-arm) in all seven dipluran species, which differs slightly from the claim of Podsiadlowski et al. (2006) who reconstructed this arm as merely shortened in *J. solifugus*. In addition, all three campodeid species show D-arm loss in *trnR* and *trnS2,* whereas in *C. lubbocki,* the D-arm of *trnC* is also truncated. The secondary structures of tRNA of the projapygoid *O. sinensis* are more similar to those of the japygoid species sampled thus far.

## Discussion

### Artifacts and the Effects of Taxonomic Sampling

Maximum likelihood methods estimate phylogenetic relations by modeling the sequence evolution (i.e., nucleotide substitution patterns) of genes to construct the gene trees (Swofford et al. 1996). Model violations, however, can cause incorrect phylogenies when the sequences evolved especially fast (leading to mutational saturation), when the evolution was not uniform across all taxa, or if the evolutionary patterns otherwise failed to fit the assumptions of the model (Rodríguez-Ezpeleta et al. 2007). Unrelated taxa with rapidly evolving, divergent genes (long branches) can group together in trees by a LBA artifact (Felsenstein 1978; Hendy and Penny 1989), especially when their genes have convergently evolved similar base compositions (e.g., a high AT content). Because of the complexity of mitochondrial genomic evolution, LBA artifacts plague the phylogenies derived from the mt genomes of arthropods (Hassanin et al. 2005; Hassanin 2006; Talavera and Vila 2011; Simon and Hadrys 2013). This problem lowers the support values at the tree nodes and explains the low bootstrap values of most of the deepest branches in our full-taxon tree of figure 1. An especially obvious LBA artifact is at the top of figure 1 where the two hemipterans (true bugs), which are universally accepted to be winged insects, appear as polyphyletic with one of their long-branch sequences, *Bemisia*, grouping with a noninsect proturan and the other bug, *Schizaphis*, grouping with an advanced, holometabolous insect (bee *Apis*), in both cases with moderately high—and highly erroneous—bootstrap support.

In this study, we used multiple approaches to minimize the systematic errors of LBA (Delsuc et al. 2003; Rodríguez-Ezpeleta et al. 2007). We improved the evolutionary models for likelihood analysis by properly partitioning the gene data, removed the eight taxa with the longest branches, and used RY coding to lessen the effects of saturation and base composition heterogeneity (Delsuc et al. 2003; Phillips and Penny 2003). We also paid special attention to whether presenting the protein sequences as amino acids gave the same results as expressing them as nucleotides (they did). However, all these different approaches failed to support the monophyly of Diplura (row B in table 3), until we included the projapygoid *O. sinensis* (rows A, D–F). We also noticed that monophyly of Diplura was recovered with low bootstrap value (18%–36%) in the tests containing only the three previously studied dipluran taxa and when both the first and third codon positions were RY-coded (row C in table 3). Although these bootstrap values are far below statistical significance, this hints that some of the signal for Diplura polyphyly was from base heterogeneity. However, the major source is from incomplete taxon sampling. As long as the projapygoid is present, excluding all the campodeoid or japygoid sequences does not disrupt this dipluran monophyly. Therefore, our results show that including Projapygoidea is the key for retrieving a monophyletic Diplura in mitogenomic analyses.

Including a large number of taxa in phylogenetic analysis is a good way to improve the accuracy of the inferred trees, but this need not mean random inclusion of as many taxa as possible (Lecointre et al. 1993; Poe and Swofford 1999; Lin et al. 2002; Zwickl and Hillis 2002; Pick et al. 2010; Dimitrov et al. 2012). In fact, our analytical tests show that three of the four new mitogenomic sequences (Campodeidae: *L. weberi*, Parajapygidae: *P. emeryanus*, and Japygidae: *Occ. japonicus*) are entirely dispensable for recovering a monophyletic Diplura. The contribution of each taxon to the accuracy of a phylogenetic tree may be different when the taxa number increases, so we suggest following the taxonomic classification for taxa selection. That is, we advocate sampling wisely, focusing on what seem to be the key subclades not yet sampled. It is more important to increase the sampling diversity than the quantity alone (Poe and Swofford 1999; Pollock et al. 2002; Lin et al. 2002; Bininda-Emonds and Stamatakis 2007).

### Phylogeny of Diplura

Our discovery that rigorously analyzed mt genomic sequences from the full range of diplurans support dipluran monophyly agrees with most of the evidence from nuclear genes and morphology (Edgecombe 2010; Giribet and Edgecombe 2012; Trautwein et al. 2012). From the viewpoint of morphology, the only evidence against monophyly of Diplura involves different ovarian structures in campodeids versus japygids (Štys et al. 1993), which according to our phylogenetic results imply reversals to ancestral-hexapod states in the Campodeidae. The abundant counterevidence, for dipluran monophyly, includes the synapomorphies summarized by Koch (2009), among which is a unique entognathous condition that differs from the entognathy of proturans and collembolans (Koch 1997; Sekiya and Machida 2011); molecular phylogeny based on nuclear rRNA genes (Luan et al. 2005); and phylogenetic analysis of nuclear PCGs (Regier et al. 2010).

The phylogenetic position of Projapygoidea within Diplura is a key issue for reconstructing their phenotypic evolution. Rusek (1982) considered Projapygoidea as a relict group of “living fossils” among diplurans in showing a combination of morphological characteristics of Campodeoidea and Japygoidea, such as structures of their cerci and lacinia. In all our analyses, the projapygoid *O. sinensis* is more closely related to Campodeoidea than to Japygoidea with high bootstrap values (table 3). This finding conflicts with previous results obtained from analysis of nuclear 18S and 28S rRNA genes (Luan et al. 2005; Gao et al. 2008) but is in accordance with the classical division of Diplura into Rhabdura (Campodeoidea and Projapygoidea) and Dicellurata (=Japygoidea) (Pages 1997). This division also found support in cladistic analysis of characters of the external morphology (Bitsch and Bitsch 2000).

The phylogenetic position of Diplura within Pancrustacea remains unclear (Luan et al. 2005; Mallatt et al. 2010; Regier et al. 2010), and mitochondrial genomes failed to provide a clear resolution of relations among the main pancrustacean groups in previous mt genomic analyses (Nardi et al. 2003; Cook et al. 2005; Carapelli et al. 2007; Chen et al. 2011). Our study likewise fails to recover the monophyly of Hexapoda or to find Diplura’s sister group. However, because its improved taxon sampling yielded dipluran monophyly, it seems to have solved one of the longstanding problems. This offers some hope that a denser sampling with more key taxa of the basal hexapods, along with better tree-reconstruction models, can resolve more pancrustacean clades in future mt genomic studies.

### tRNA Truncation

The state of the D-arm in dipluran tRNA reflects the phylogeny of Campodeidae (arrows in fig. 4). Loss of this arm in *trnR* and *trnS2* is an apparent autapomorphy of the Campodeidae. Members of this family have the largest number of truncated tRNAs (fig. 4), which suggests that they are more derived than are japygoid and projapygoid species. Within Campodeidae, *C. lubbocki* furthered the trend with its unique loss of the D-arm in *trnC*. The *trnS1* of all seven diplurans lacks the D-arm; however, the remnant loops of *O. sinensis* (12 bp) and of the japygoid species (11 bp for *P. emeryanus*, 10 bp for *Occ. japonicus* and 9 bp for *J. solifugus*) are larger than those of the campodeid species (5 bp for *C. fragilis* and *C. lubbocki*, 4 bp for *L. weberi*), again indicating more loss in campodeids. The projapygoid *O. sinensis* is similar to the three japygoid species in its tRNA secondary structure (fig. 4) but is sister to three campodeid species on our phylogenetic trees, which suggests that it retains the ancestral state of dipluran tRNA structure.

It is noteworthy that all the dipluran tRNA truncations involve loss of their DHU arms, whereas the truncation in tRNAs of nematodes (Wolstenholme et al. 1987), arachnids (Masta and Boore 2008), proturans (Chen et al. 2011), and gall midges (Beckenbach and Joy 2009) involves primarily the TΨC arm. For further comparison, the 18 tRNAs of the proturan *Si. erythranum* show truncated secondary structures, but only three of them involve loss of the DHU arm (*trnC*, *trnY,* and *trnS1*) (Chen et al. 2011). Compared with the cases of severe truncation of tRNA genes mentioned above, the tRNA truncations of Diplura are less remarkable. This may be why tRNA truncations in Diplura are phylogenetically informative, whereas not so in animals with severely truncated tRNA, which seem to have lost phylogenetic signal through saturation.

## Supplementary Material

Supplementary files S1–S3 are available at *Genome Biology and Evolution* online (http://www.gbe.oxfordjournals.org/).
